# Characterization of the Early Proliferative Response of the Rodent Bladder to Subtotal Cystectomy: A Unique Model of Mammalian Organ Regeneration

**DOI:** 10.1371/journal.pone.0047414

**Published:** 2012-10-12

**Authors:** Charles C. Peyton, David Burmeister, Bryon Petersen, Karl-Erik Andersson, George Christ

**Affiliations:** 1 Department of Urology, Wake Forest Baptist Medical Center, Winston Salem, North Carolina, United States of America; 2 Institute for Regenerative Medicine, Wake Forest University, Winston Salem, North Carolina, United States of America; University of Minnesota Medical School, United States of America

## Abstract

Subtotal cystectomy (STC; surgical removal of ∼75% of the rat urinary bladder) elicits a robust proliferative response resulting in complete structural and functional bladder regeneration within 8-weeks. The goal of these studies was to characterize the early cellular response that mediates this regenerative phenomenon, which is unique among mammalian organ systems. STC was performed on eighteen 12-week-old female Fischer F344 rats. At 1, 3, 5 and 7-days post-STC, the bladder was harvested 2-hours after intraperitoneal injection of bromodeoxyuridine (BrdU). Fluorescent BrdU labeling was quantified in cells within the urothelium, lamina propria (LP), muscularis propria (MP) and serosa. Cell location was confirmed with fluorescently co-labeled cytokeratin, vimentin or smooth muscle actin (SMA), to identify urothelial, interstitial and smooth muscle cells, respectively. Expression of sonic hedgehog (Shh), Gli-1 and bone morphogenic factor-4 (BMP-4) were evaluated with immunochemistry. Three non-operated rats injected with BrdU served as controls. Less than 1% of cells in the bladder wall were labeled with BrdU in control bladders, but this percentage significantly increased by 5-8-fold at all time points post-STC. The spatiotemporal characteristics of the proliferative response were defined by a significantly higher percentage of BrdU-labeled cells within the urothelium at 1-day than in the MP and LP. A time-dependent shift at 3 and 5-days post-STC revealed significantly fewer BrdU-labeled cells in the MP than LP or urothelium. By 7-days the percentage of BrdU-labeled cells was similar among urothelium, LP and MP. STC also caused an increase in immunostaining for Shh, Gli-1 and BMP-4. In summary, the early stages of functional bladder regeneration are characterized by time-dependent changes in the location of the proliferating cell population, and expression of several evolutionarily conserved developmental signaling proteins. This report extends previous observations and further establishes the rodent bladder as an excellent model for studying novel aspects of mammalian organ regeneration.

## Introduction

Although regeneration *per se* occurs throughout the animal kingdom, there are large disparities in the degree of regeneration observed between species (e.g., amphibian versus mammalian) let alone amid organs (e.g., liver versus kidney).[1], [2], [3] The extensive attention focused on regenerative medicine is understandable given the enormous potential for repair and/or replacement of old, damaged or diseased cells, tissues and organs; such as the diseased and dysfunctional bladders that are the subject of this report. To date, the vast majority of investigations have focused on the utility of exogenous stem cells and biomaterials, either alone or in combination, to heal, repair and/or restore function to damaged cells, tissues and organs.[4], [5], [6], [7] Again, it is important to emphasize that this work has been conducted with incomplete knowledge of how the regenerative process actually works to restore bladder function in the absence of exogenous manipulation/assistance. In this scenario, there is a significant knowledge gap about how endogenous organ regeneration works in adult mammals. This factual ignorance represents a clear barrier to more rapid scientific progress, and thereby, inhibits clinical translation.

In this regard, important guidance about the characteristics and boundary conditions of organ regeneration can be derived from the extensive existing literature on the liver. In fact, the liver has an extensive regenerative potential in response to tissue loss.[8], [9] Liver re-growth is a systematic process that replaces the lost or damaged tissue through an orchestrated series of events, first introduced into the scientific literature in 1931 by *Higgins and Anderson*, who pioneered the 2/3 partial hepatectomy rat model.[10] However, the concept has been known for thousands of years dating back as far as ancient Greek mythology and the story of Prometheus. The liver is very efficient in repairing or regenerating its mass, which occurs as a direct result of the proliferation of all the existing cells from the remaining liver remnant, but is mainly driven by mature hepatocytes, which will re-enter the cell cycle to restore the liver.[11], [12] The entire process, which is usually referred to as regeneration, is completed within a couple weeks, depending upon the mammalian species. However, the process is more accurately termed compensatory hyperplasia.[13]

These well established observations regarding liver re-growth stand in contrast to rodent bladder regeneration, which occurs over a longer time frame (8 weeks rather than 2 weeks), and moreover, results in a regenerated bladder that structurally and functionally is essentially identical to the native bladder which it replaced.[14], [15], [16] More specifically, the bladder capacity and bladder wall thickness (as well as the presence of all three layers; urothelium, muscularis propria and lamina propria) of the regenerated bladder are indistinguishable from the previous native bladder, and moreover, the animals are entirely continent.[14] To our knowledge, bladder regeneration therefore holds a unique position with respect to its regenerative potential, as there is no other mammalian organ capable of this type of regeneration.

The rather remarkable regenerative capacity of the bladder has been recognized in rodents, canines and humans for decades.[6], [17], [18], [19], [20] This insight, coupled with the limitations and complications associated with using intestinal bowel segments has led to pursuit of new treatment options for bladder augmentation and reconstruction, and recently culminated in the development of autologous cell-seeded polymer scaffolds as an alternative to traditional cystoplasty and/or neobladder techniques.[4] However, clinical experience with this technology is limited and not yet ready for wide dissemination, pointing to the need for further investigations into the potential applications of tissue engineering/regenerative medicine applications to bladder reconstruction.[7] To this end, the goal of this initial investigation was to gain improved understanding of the molecular and cellular mechanisms of bladder regeneration by studying the spatiotemporal characteristics of the proliferative response to subtotal cystectomy (i.e., STC; removal of ∼75% of the bladder) during the early stages of bladder regeneration in a rodent model *in vivo*. The current findings extend previous work [14] and point toward the utility of the urinary bladder as an important and unique model of mammalian organ regeneration.

## Materials and Methods

### Animals and ethics statement

The animal protocol was approved by the Animal Care and Use Committee of Wake Forest University and carried out with strict adherence to the guidelines set fourth. All surgeries were performed under appropriate anesthesia with postoperative pain medication. A total of 18 female Fisher F344 rats weighing 154–189 g underwent subtotal cystectomy (STC), with three animals dying after surgery due to surgical complications. Three animals were removed from the study due to technical artifacts that prevented obtaining accurate histologic tissue sections (e.g., peritoneal urine leak and bladder blood clot). The remaining 12 animals were utilized in this study, with 3 age-matched control animals run in parallel (i.e., no cystectomy).

### Trigone Sparing Cystectomy

Animals were anesthetized with 2–5% isoflurane chamber induction and maintained with 2–3% via mask inhalation with spontaneous breathing. The lower abdomen was shaved and decontaminated with betadine. A low midline abdominal incision was made with sufficient exposure to identify and deliver the bladder. After bladder exposure, two stay sutures were placed just above the ureterovesical junction (UVJ) using 6–0 polyglycolic acid sutures for traction and alignment. The bladder dome (approximately 70–80% of the whole bladder) superior to the UVJs was then removed, sparing the trigone and ureters. The open bladder was then sutured closed in a continuous fashion using one of the original stay sutures and ensuring adequate coaptation of the anterior and posterior bladder walls as previously described.[14] Care was taken to avoid excessive pulling force or suturing over the UVJs. Once the remaining bladder was repositioned into the abdomen, the abdominal wall and skin were closed in two layers using 3–0 polyglyocolic acid sutures.

### BrdU Injections and Euthanasia

Two hours prior to sacrifice, rats were injected intraperioneally with 50 mg/kg of 5-Bromo-2′-deoxyuridine (Sigma-Aldrich, product no. B5002). Three animals were injected and sacrificed at 1, 3, 5, and 7-days after STC using CO_2_ asphyxiation followed by cervical dislocation. An additional three age-matched control animals were also injected and euthanized.

### Histological analysis

**Figure 1:**
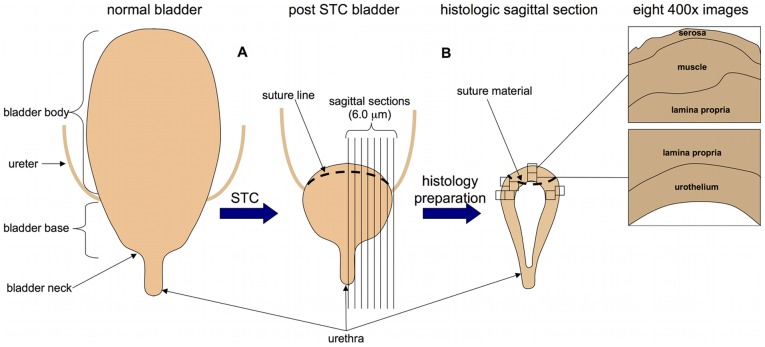
Illustration of STC and serial sectioning for immunochemical and histologic analysis of regenerating bladder tissue. (**A**) The bladder body is excised leaving the trigone and ureterovesical junction. (**B**) At 1, 3, 5 and 7 days post-STC remaining bladder is harvested and serial sections are prepared for analysis of the 4 distinct bladder wall layers using adjacent 400x images.

**Figure 2:**
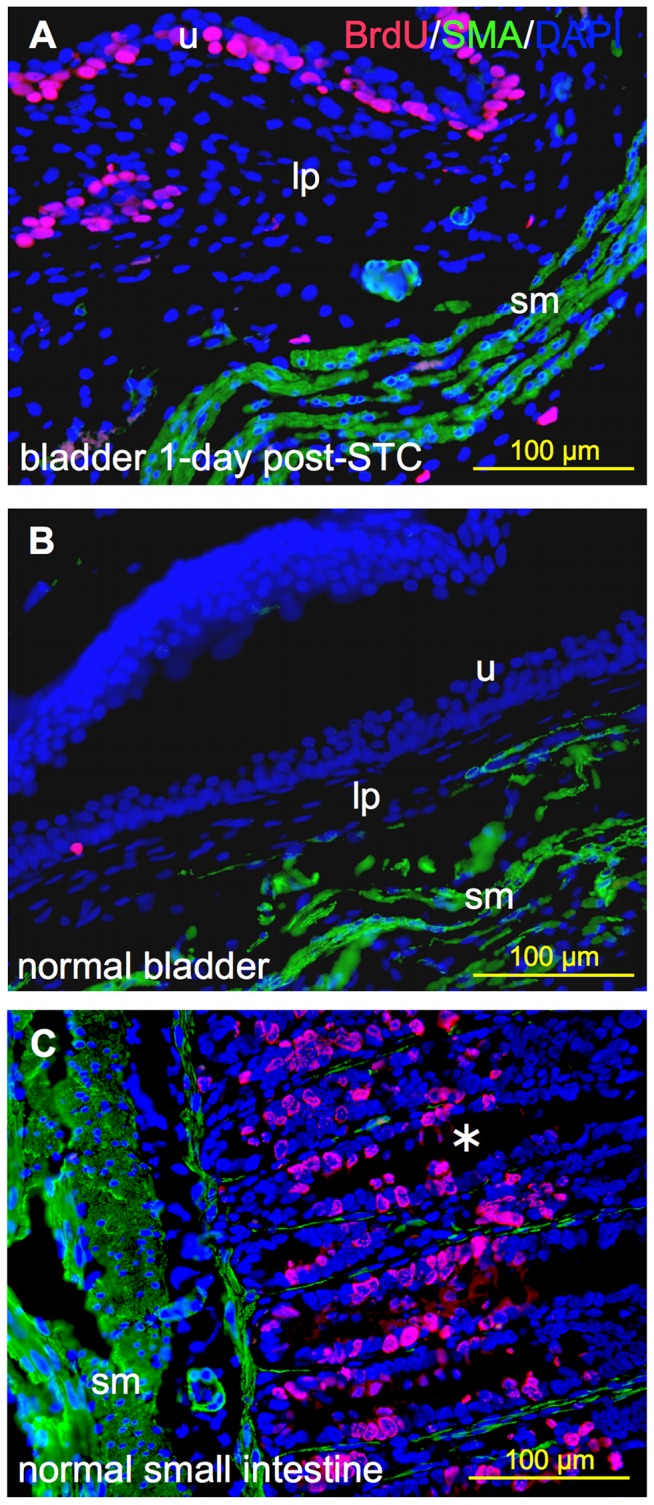
Comparison of BrdU-labeling in regenerating bladder versus normal native bladder and small intestine. Representative 400x immunoflurescent images showing BrdU-labeled cells (pink) relative to the distribution of smooth muscle alpha-actin immunostaining (SMA, green) and nuclear staining with DAPI (blue). (**A**) Regenerating bladder 24-hrs post-STC, (**B**) native control bladder and (**C**) normal small intestine (positive control). **(B)** and **(C)** were derived from the same animal. Note the expected higher cellular proliferation rate observed in regenerating bladder and small bowel relative to the native bladder. (**u**) urothelium; (**lp**) lamina propria; (**sm**) smooth muscle; and (*) denotes an intestinal crypt.

After sacrificing the animals, the abdomen was reopened and the bladder identified. The remaining bladder from the site of STC closure to the distal trigone was explanted, fixed in 10% buffered formalin overnight, processed and embedded in paraffin. Serial 6 µm sagittal tissue sections were sliced along the cranial-caudal axis of the bladder. Figure 1 illustrates STC and bladder sectioning. Animals that did not undergo STC and received BrdU prior to sacrifice served as controls for all immunohistochemisty. Small bowel was also harvested from control animals as a positive control for BrdU uptake due to its highly proliferative epithelium (Fig. 2). Standard hematoxylin and eosin staining was performed to assess cell morphology and inflammation.

Fluorescent immunohistochemistry for each time point was performed as follows: heat activated antigen retrieval was performed in 0.01 molar, pH 6.0 citrate buffer. After cooling for 30–60 minutes, the slides were washed in PBS, followed by 15-minute incubation with Serum-Free Protein Block (Dako, ref. no X0909). For quantitative analysis of DNA-replicating cells, double stains were performed for the following primary antibodies/incubation times: anti-BrdU (Abcam; ref. no. ab8152, mouse monoclonal, 1∶50 dilution) for 5 hours at room temperature and anti-SMA (Abcam; ref. no. ab5694, rabbit monoclonal, 1∶200 dilution) overnight at 4°C. Following primary antibody incubation, slides were washed with PBS and a second protein block for 5 minutes was performed. Secondary antibodies Texas Red anti-mouse monoclonal (Vector; ref. no. TI-2000) and Fluorescein anti-rabbit polyclonal (Vector; ref. no. FI-1000, 1∶250 dilution) for 30 minutes at room temperature in a dark chamber recognized BrdU and SMA primary antibodies, respectively. For confirmation of BrdU-labeled cell location a subset of additional double stains were performed using the following antibodies/incubation times: anti-BrdU primary and secondary antibodies as described above with anti-cytokeratin (Dako; ref. no. M3515, mouse monoclonal, 1∶50 dilution) for one hour at room temperature or anti-vimentin (Santa Cruz, ref. no. sc-7557, goat monoclonal 1∶50 dilution) for one hour at room temperature. Fluorescein anti-mouse and anti-goat polyclonal antibodies (Vector; ref. no. FI-2000 and FI-5000, 1∶250 dilution) for 30 minutes at room temperature in the dark recognized cytokeratin and vimentin primary antibodies, respectively. For all slides, nuclei were nonspecifically counterstained with 4′–6′diamidino-2-phenylindole (DAPI) mounting medium (Vector; ref. no. H-1200) and coverslipped.

Bright field immunohistochemistry probing Shh, Gli-1, and bone morphogenic protein-4 (BMP-4) was performed similarly. Following antigen retrieval, endogenous peroxidase activity was blocked using 10∶1 dilution of 70% methanol and 30% hydrogen peroxide. Primary antibodies against Shh (Novus; ref. no. NBP1-42608, rabbit polyclonal, 1.5 µg/ml dilution), Gli-1 (Sant Cruz; ref. no. sc-20687, rabbit polyclonal, 1∶200 dilution), and BMP-4 (Abcam; ref. no. ab39973, rabbit polyclonal, 1∶100 dilution) were incubated 1 hour at room temperature. Following primary antibody incubation and protein block, slides were treated for 30 minutes with secondary antibody biotinylated anti-rabbit (Vector; ref. no. BA-1000) and ABC reagent (Vector; ref. no. PK-7100). Shh and BMP-4 slides were then incubated with diaminobenzidine (DAB) chromagen (Vector; ref. no. SK-4100) for 5–10 minutes, except for BMP-4 slides which were incubated with Nova Red (Vector; ref. no. SK-4805) for 5–10 minutes. Lastly, slides were lightly counterstained with hematoxylin and mounted.

### Microscopy Quantification

**Figure 3:**
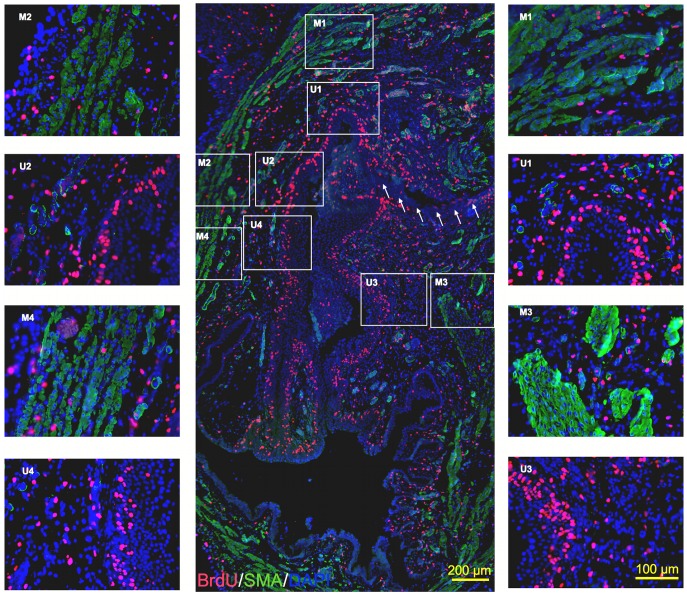
Global and robust distribution of proliferating cells at 3-days post-STC. (**A**) 100x center image depicts a sagittal bladder section oriented from site of STC (top of image) moving towards the trigone (bottom). **Arrows** indicate evidence of suture line used to close the bladder. (**M1/U1 – M4/U4)** Representative adjacent images at 400x magnification used for cell quantification. BrdU (red), SMA (green), DAPI (blue).

**Figure 4:**
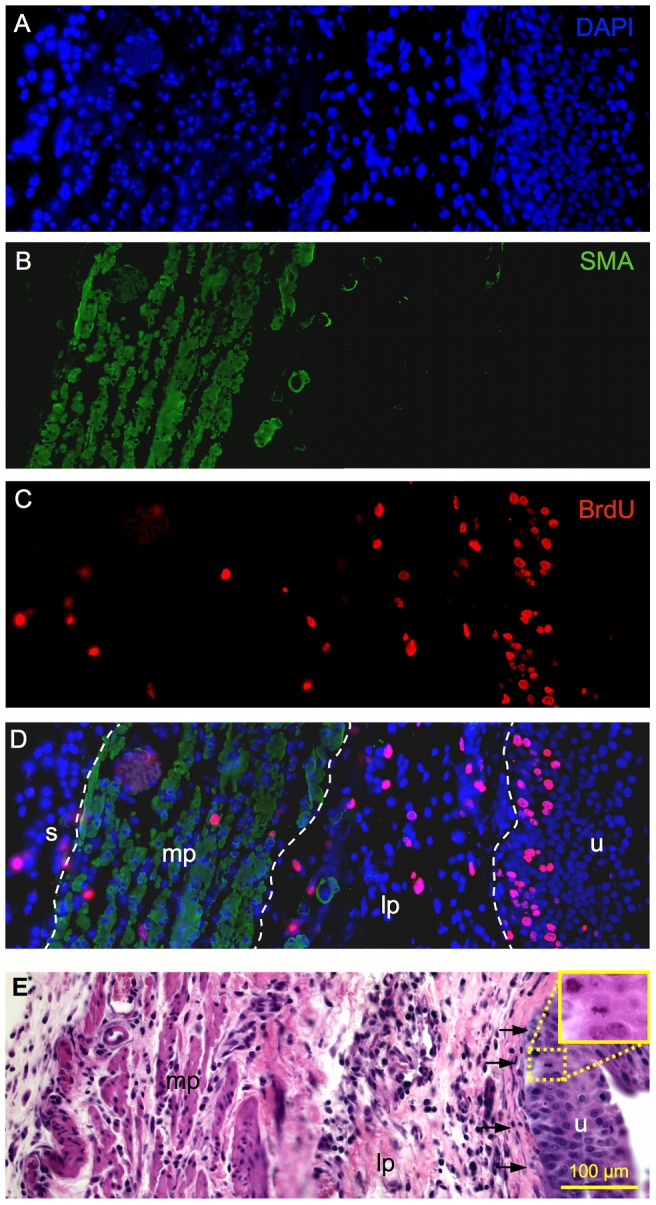
Image construction and cell localization method. Representative transmural 400x images from the regenerating bladder wall at 3-days post-STC. Spectrally unmixed images from the same tissue section illustrate (**A**) nuclear DAPI staining, (**B**) SMA, and (**C**) BrdU labeled cells. (**D**) The composite overlay image used for cell counts and location is also shown. White dashed lines distinguish among the distinct bladder wall layers. (**E**) Serially sectioned 400x H&E image showing basal layer of the lamina propria (**arrows**) and 630x inset demonstrating a urothelial mitotic figure. (**mp**) muscularis propria, (**lp**) lamina propria, (**u**) urothelium, (**s**) serosa.

Fluorescent microscopy was used to examine the number of BrdU-labeled cells and their location within the tissue layers of the bladder at each time point after STC. A Leica (model no. DM4000B) upright microscope with ImagePro software 6.3 (Media Cybernetics, Bethesda, MD) was used to take eight 400x images of each tissue section per animal. Suture material at the STC closure site was used to orient the sagittal tissue sections, and systematic images were taken focusing on the upper half of the full tissue section (e.g., closest to the plane of bladder excision). Four sets of paired images spanning the width of the bladder wall were taken for each animal (Fig. 3) and captured a transmural picture of the bladder wall including all four layers: urothelium, lamina propria (LP), muscularis propria (MP) and serosa (Fig. 4D).

To quantify BrdU-labeled cells, fluorescent staining for smooth muscle actin (SMA) helped define the MP layer. The LP was defined as the area between the basal lamina underlying the urothelium and the MP (denoted with SMA). The area beyond the MP was considered serosa. Figure 4 illustrates adjacent, merged 400x transmural bladder wall composite image construction using different microscopic filters (DAPI, GFP, Texas Red) specific to secondary antibody immunofluroescence. As can be seen, composite images highlight the location of BrdU-labeled cells and serial H&E sections were used for morphological reference (eg, basal lamina, Fig. 4E). A dashed line was inserted into each image to distinguish the urothelium, LP, MP, and serosa of the bladder wall (Fig. 4D). A non-biased, third-party reviewer counted both the total number BrdU-labeled and the total number of cells via DAPI positive nuclei in each layer of the bladder wall. Three animals were analyzed at 1, 3, 5, and 7-days post-STC, along with three normal control bladders, providing 24 images per group. Cell counts were performed for a total of 120 images.

To further confirm the location BrdU-labled cells and better define their cell type, an Olympus FV1000 laser scanning confocal microscope with a 60x oil objective and Fluoview software (Olympus) allowed for high power, reconstructed z-stack examination of a subset of BrdU-cytokeratin, BrdU-vimentin and BrdU-SMA dual fluorescence images. Cytokeratin defined the urothelium, vimentin highlighted LP interstital cells, and SMA identified MP.

### Statistical Analysis

Statistical analysis was performed using GraphPad Prism software. For BrdU-labeling quantification a one-way ANOVA was used compare the total percentage of labeled cells for each time point, and a two-way ANOVA with Bonferroni post-hoc testing was used to compare the time-dependent location changes in BrdU-labeled cells. A *p*-value of less that 0.05 was considered significant. All results were expressed as the mean ± standard error of the mean.

## Results

### BrdU Quantification

BrdU labeling was used to evaluate the spatiotemporal characteristics of the cellular response to STC. Figure 1 provides a schematic diagram of the bladder tissue sampling method employed, while Figure 2 documents the utility of the BrdU-labeling method. As illustrated, fewer than 1% of cells were labeled with BrdU in native control bladders. In contrast, abundant BrdU-labeling was apparent as early as 1-day post-STC. Moreover, as expected, considerable BrdU-labeling was observed in the epithelial layer of the small intestine from the same control animal; this latter observations is consistent with previously published observations of an intestinal epithelial cell turn-over rate of approximately 15–30% using the same cell labeling techniques (Fig. 2).[21], [22]

**Figure 5:**
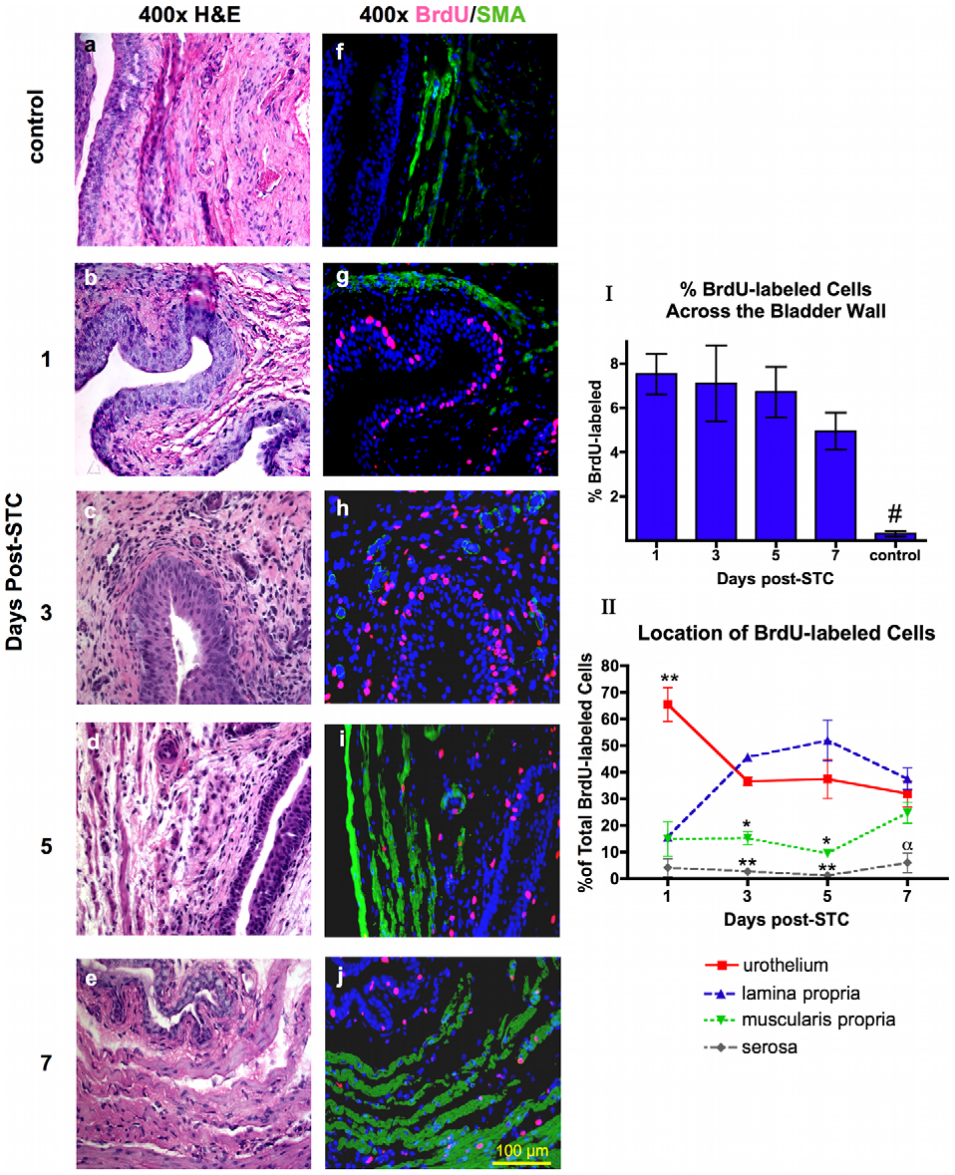
Time-dependent changes in the location of the proliferating cell population in the bladder wall layers post-STC. Representative H&E sections (**a-e**) with corresponding serial immunofluorescent images (**f-j**). (BrdU-pink, SMA-green, DAPI-blue). Note the absence of BrdU-labled cells in control (**f**) in contrast to the abundant BrdU-labeled cells that are obvious in the urothelium at 1-day post-STC (**g**), while the percent of BrdU-labeled cells changes to reveal a greater percent of labeled cells in the LP at 3 and 5-days post-STC (**h, i**). By 7-days post-STC the distribution of BrdU-labeled cells is similar among the urothelium, LP, and MP (**j**). Quantification of numerous images was used for graphical depiction. (**Ι**) All bladders subjected to STC had a significantly higher percentage of BrdU-labeled cells than control, (one-way ANOVA, **#** - *p* = 0.004). (**ΙΙ**) The location of BrdU-labeled cells (expressed as a percentage of total BrdU cells) is significantly different in the distinct bladder wall layers during the first week of bladder regeneration. Note that serosal BrdU labeling remained significantly less than urothelium at all time points, less than LP at 3, 5 and 7-days, and less than MP at 7-days. MP was significantly less than urothelium and LP at 3 and 7-days. (Two-way ANOVA, ** - *p*<0.001, * - *p*<0.01, α - *p*<0.05). N = 3 animals at each time point.

Figure 3 is a representative montage of immunofluorescent microscopic images demonstrating the prominent, global proliferative response observed 3-days post-STC. Spectrally unmixed and merged adjacent microscopic images are shown in Figure 4, highlighting the method for quantification of BrdU-labeled cells in the distinct layers of the bladder wall. As shown in Figure 5, the percentage of BrdU-labeled cells was significantly higher in the bladder wall after STC compared to control bladders at all time points (*p* = 0.004), and, although not significant, there was a tendency for the percent of BrdU-labeled cells to decrease with time after STC (Fig. 5-Ι).

In order to compare the impact of STC on BrdU labeling in the various layers of the bladder wall, the amount of BrdU-labeling was expressed as a percentage of the total number of BrdU-labeled cells throughout the bladder wall (Fig. 5-ΙΙ). As illustrated, at 1-day post-STC the percentage of BrdU-labeled cells in the urothelium was significantly elevated compared to LP, MP and serosa (65.4±6.3, versus 15.6±1.1, 14.8±6.4, and 4.1±3.4% respectively, *p*<0.001). Interestingly, a time-dependent shift in the location of BrdU-labeled cells was noted. More specifically, at 3 and 5-days post-STC the vast majority of BrdU-labeled cells (80–90%) are found in the urothelium (36.5±1.6 and 37.4±7.2% at 3 and 5-days, respectively) and LP (45.5±0.8 and 51.9±7.6% at 3 and 5-days, respectively). Significantly fewer labeled cells were detected in the MP and serosa at 3 and 5-days post-STC (15.2±2.4 and 9.5±0.3% for MP; 2.6±0.3 and 1.1±0.5% for serosa, respectively, versus 36.5±1.6 and 37.4±7.2% for urothelium; 45.58±0.8 and 51.9±7.6% for LP, respectively, *p*<0.01). At 7-days post-STC, the percentage of BrdU-labeled cells was similar across the urothelium, LP and MP (31.8±4.8%, 37.5±4.0%, 24.7±3.9% respectively, *p*>0.05). The proportion of labeled cells in the serosa remained relatively low at all time points (1.1±0.4% to 5.9±3.6%).

**Figure 6:**
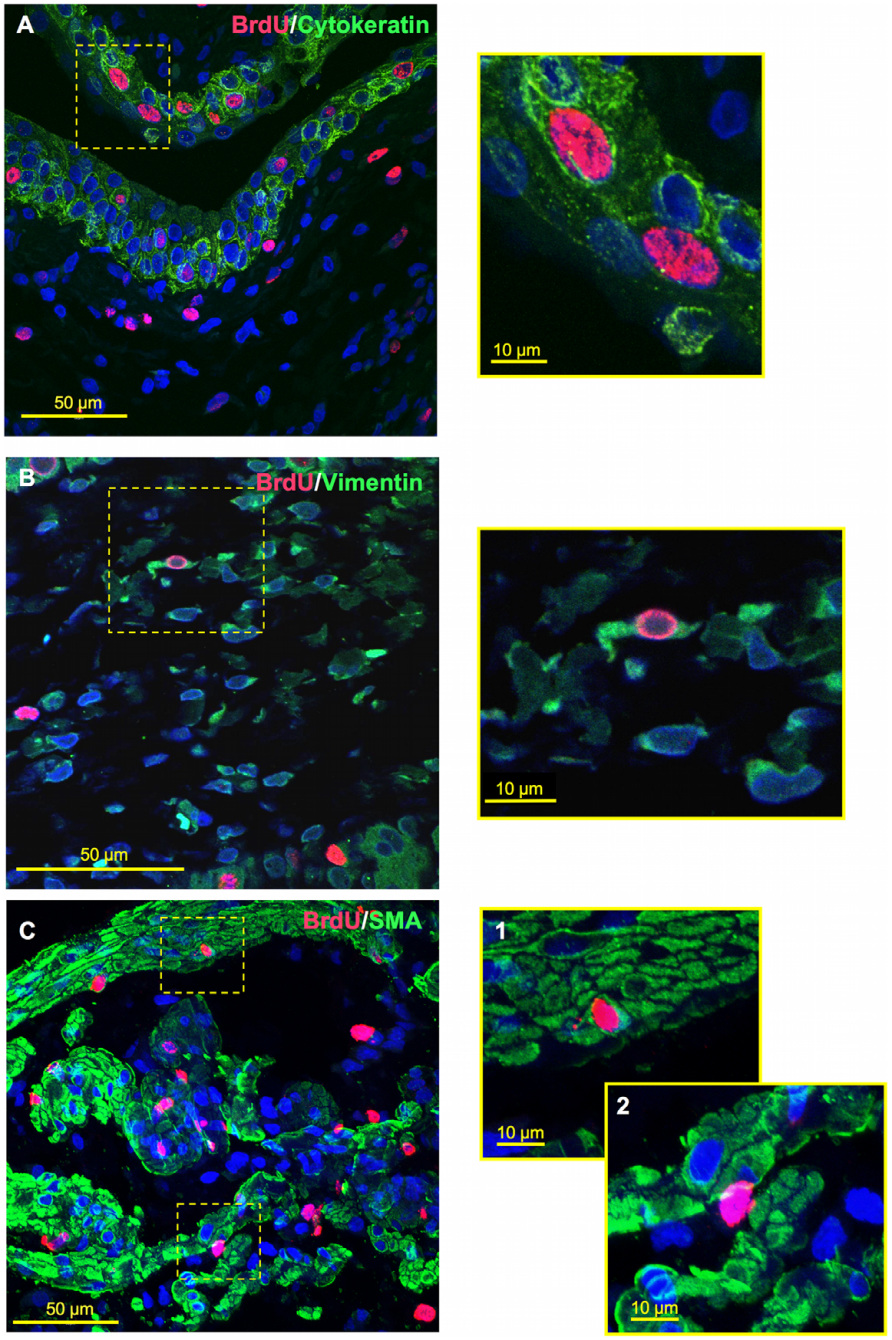
Co-labeling of BrdU expressing cells with cell-specific markers for the urothelium, MP and LP. Confocal z-stack reconstruction imaging was performed at 600x magnification, where offset pictures are digitally zoomed. (**A**) BrdU-cytokeratin co-labeling within urothelium at 3-days post-STC. (**B**) BrdU-vimentin co-labeling with LP at 3-days post-STC. (**C**) 7-days post-STC BrdU-SMA co-labeling was also observed within the MP (**C-1**), but was relatively rare. BrdU-labeled cells within the MP were more commonly observed between smooth muscle cells as well as smooth muscle bundles (**C-2**).

**Figure 7:**
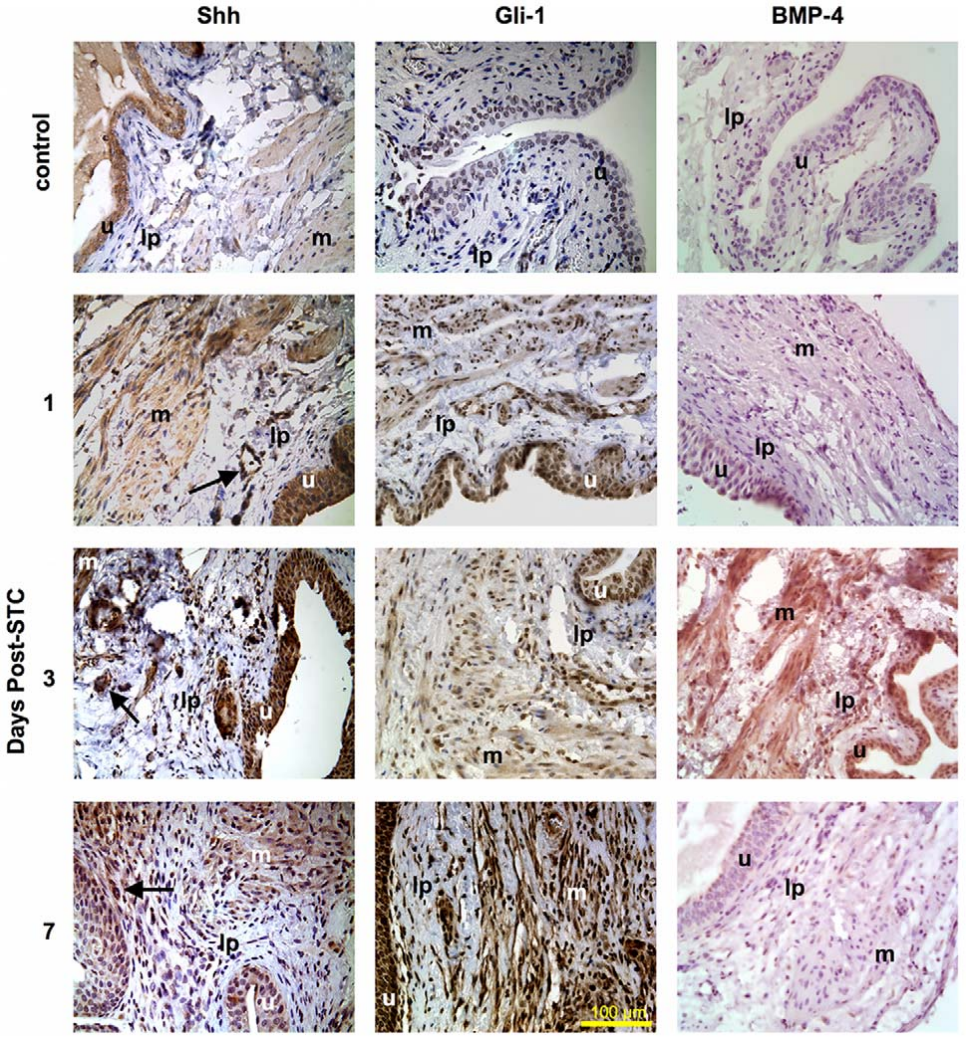
Contribution of the hedgehog pathway to bladder wall regeneration post-STC. Immunostaining for Shh and its downstream effectors Gli-1 and BMP-4 in a native control bladder (top panel), as well as regenerating bladder at 1, 3 and 7 days post-STC (400x magnification). The far left column demonstrates moderate baseline Shh positivity in the control bladder with apparently increased staining within the urothelium and MP at 1, 3 and 7 days after STC. Vessels also appear to express Shh (arrows) during bladder wall regeneration. The middle column reveals that Gli-1 expression also appeared enhanced at 1, 3 and 7 days post-STC in the urothelium, LP and MP. The right column demonstrates negative staining for BMP-4 in the control bladder and apparently enhanced BMP-4 at 3-days post-STC. (**Shh**) sonic hedgehog; (**BMP-4**) bone morphogenic protein 4; (**u**) urothelium; (**lp**) lamina propria; (**m**) muscularis propria.

Co-labeling experiments were also performed to provide further detail about the location and nature of the BrdU-labeled cells in the various layers of the bladder wall following STC. Confocal microscopy of dual fluorescently-labeled tissue sections was performed. Figure 6 displays a representative example of a collection of images taken from bladder tissue excised at 3-days post-STC (Fig. 6-A and B) and 7-days post-STC (Fig. 6-C). As illustrated, BrdU-labeled cells within the urothelium were consistently co-labeled with cytokeratin – a prominently expressed epithelial cytoskeleton protein. BrdU-labeled cells in the LP occasionally co-labeled with vimentin, a common intermediate filament marking interstitial cells of the bladder wall.[23], [24] In addition, we noted BrdU-SMA co-labeling, as seen in Figure 6-C1; although this was relatively rare overall ranging from 1.6±0.37 to 3.3±1.1% of BrdU-labeled cells within the MP. More commonly BrdU-labeled cells within the MP were found between SMA-positive smooth muscle cells and bundles (Fig. 6-C2).

### Shh, Gli-1, BMP-4 Expression

**Figure 8:**
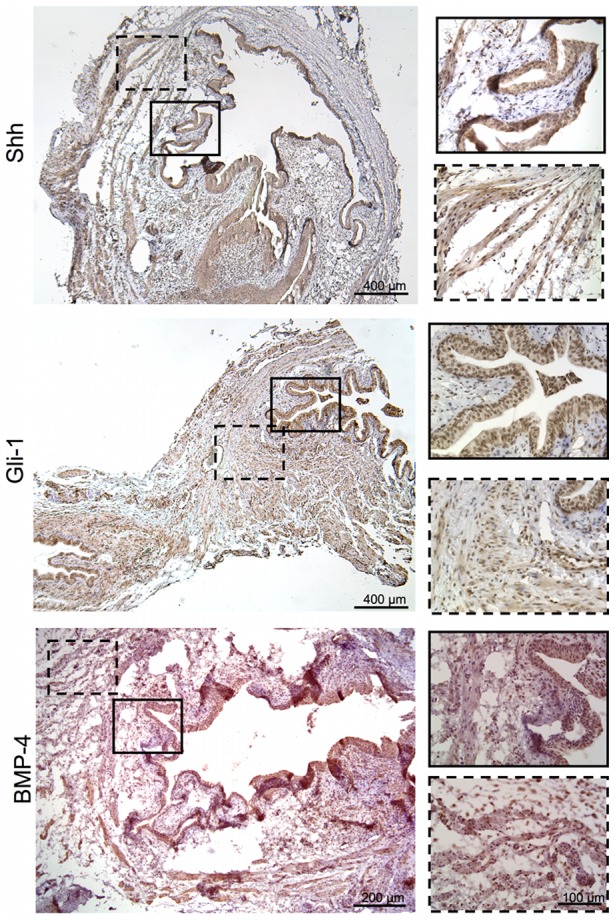
Shh, Gli-1 and BMP-4 expression at 3-days post-STC throughout the bladder wall. Low power views (Shh and Gli-1 at 50x, BMP-4 at 100x) confirm the transmural expression of these evolutionarily conserved signal modulators and their enhancement at 3-days post-op. All three signals were clearly noted in the urothelium (solid line), lamina and muscularis propria (dash line), as highlighted in the 400x insets.

Immunohistochemical staining for Shh and its downstream effectors Gli-1 and BMP-4 [25] was also performed to investigate the potential contribution of this evolutionally conserved developmental pathway to bladder regeneration post-STC. As shown in Figure 7, there was evidence for baseline Shh and Gli-1 expression in the native control bladders – most notably in the urothelium, but positive staining was also evident in the smooth muscle/stroma. In addition, these preliminary studies revealed evidence for up-regulation of Shh, Gli-1 and BMP-4 expression in the week after STC in multiple layers of the bladder wall, as seen in the low power views shown in Figure 8. More specifically, apparently enhanced Shh and Gli-1 expression was visible within the urothelium, LP and smooth muscle after STC. However, Shh was most notable at 3-days post-STC, especially within the urothelium. Conversely, Gli-1 expression appeared maximal at 7-days post-STC, and strongly highlighted the urothelium, LP and smooth muscle. Of note, vessels within the LP expressed Shh after STC at all time points. BMP-4 expression was not obvious at 1-day post-STC, but appeared to increase throughout the bladder wall at 3 and 5-days post-STC (5-day not shown). By 7-days post-STC BMP-4 expression appeared to subside.

## Discussion

A previous report documented a robust proliferative remodeling of the bladder wall following removal of ∼75% of the rat bladder, resulting in complete structural and functional bladder regeneration within 8 weeks.[14] As far as we are aware, this regenerative response is unique to the bladder in mammals. The goal of this investigation was to gain additional mechanistic insight into the spatiotemporal characteristics of this cellular response during the early stages (first week) of regeneration. Specifically, we evaluated the proliferative cellular response during the first week following STC using fluorescent BrdU cell labeling, a label-retaining technique that has been utilized to investigate a variety of stem/progenitor cell niches including liver, kidney, intestine, and previous bladder injury models.[26], [27], [28], [29], [30], [31], [32], [33]

A literature review reveals several studies that have characterized increased urothelial proliferation in response to injury (e.g., bacteria, cryogenic ablation, or outlet obstruction).[27], [31], [34] These investigations have highlighted a variety of transcriptional factors that may play an important role in the reconstitution of bladder wall cells, including evolutionarily conserved participants in hedgehog signaling, such as sonic hedgehog (Shh), Gli-1, and bone morphogenetic protein-4 (BMP-4).[31], [33], [34] Additionally, recent studies have attempted to identify bladder-specific progenitor cells that may, at least in part, participate in the observed functional recovery after injury.[35], [36]

Consistent with these observations, we report here that under normal conditions the bladder urotheilum has a low proliferative rate (Figs. 2 & 5), but is clearly capable of rapid cell turnover following injury.[37] More specifically, our study revealed that baseline urothelial cell turnover rate in the normal bladder (non-cystectomized control bladders) is very low, but greatly augmented following injury. In fact, we observed BrdU-labeling in less than 1% of all cells in control bladders (Figs. 2 & 5). Moreover, 24 hours post-STC, 7.5±0.9% of all cells (transmural) expressed the BrdU-label, with and 65±6.3% of these in the urothelium (Fig. 5). As illustrated in Figure 2, tissues derived from the *same control animal* demonstrate that the low levels of DNA synthesis/proliferation in the native bladder urothelium stand in contrast to much greater degree of BrdU labeling observed in the regenerating urothelium at 1-day post-STC, as well as the higher proliferation expected of normal gastrointestinal epithelium from the same animal.

At 1-day post-STC significantly fewer BrdU-labeled cells are present in the MP and LP, as the urothelium accounts for ∼65% of the BrdU-labeling. After this initial burst of urothelial proliferation there is a gradual shift in the location of BrdU labeling to other layers in the bladder wall. Specifically, at 3 and 5-days post-STC, there is a significant increase in the extent of BrdU labeling in the LP such that approximately 80–90% of all labeled cells are contained within these two tissue layers. However, at 7-days post-STC the percentage of BrdU-labeled cells were equivalent across the urothelium, LP and MP (Fig. 5).

In terms of identifying the nature of the BrdU-labeled cells, we can confirm that differentiated cells in the urothelium, MP and LP contribute to the overall proliferative response. For example, urothelial cells are proliferating given the morphologic characteristics and co-labeling of these cells with cytokeratin (Fig. 6). In addition, vimentin positive, presumptive interstitial cells of the LP have been of considerable interest in recent literature, and changes in interstitial cell distribution and morphology have been noted in other models of bladder dysfunction.[23], [24] Co-labeling of BrdU and vimentin positive, stellate-shaped cells within the LP suggests that these may be interstitial cells; however, we observed many BrdU-positive cells that did not co-label with vimentin. Likewise, although we observed co-labeling of SMA and BrdU, the majority of labeled cells within muscle bundles did not co-label with SMA, suggesting that another cell type is also activated. In short, the BrdU-labeled progenitor cell(s) that may be driving, at least in part, the robust proliferation observed during the first week post-STC has yet to be identified and will be the subject of future experiments.

It is interesting to compare our findings with that of other well-established models of mammalian organ regeneration, specifically, liver regeneration.[2], [9], [38] BrdU labeling has been repeatedly used to demonstrate the liver's capacity to regenerate. After partial hepatectomy, many normally quiescent hepatocytes begin DNA replication within 24 hrs, and this response gradually decreases within 4 to 10 days. Herein we report only 6–8% of all bladder cells enter the cell cycle at 24 hrs post-STC, as opposed to approximately 90% after partial hepatectomy.[28] Similar to the liver, bladder regeneration may be dependent on existing cell populations within the organ. Additionally, similar to the spatiotemporal changes in cell proliferation we observed in the bladder, the liver also displays time-dependent changes in maximal cell proliferation of the four major cell types within the liver where 30% of hepatocytes begin proliferating within the first 24-hours follow by non-parenchymal biliary ductular, Kupffer and Ito cells (20% at 2-days), and finally sinusoidal endothelial cells (10% at 4-days).[2], [9], [28]

In summary, bladder regeneration appears to be a process that shares common features with both liver regeneration (robust and sequential proliferation of cell types) and limb regeneration (30–90 day time frame and complete structural and functional restoration). Given the relatively complete structural and functional regeneration observed in the liver, it may more closely resemble blastema formation than liver regeneration. More specifically, liver regeneration is characterized by a strictly/largely hyperplastic response, while the salamander regenerates a limb via blastema formation. The blastema, in turn, may be the result of dedifferentiation, transdifferentiation, expansion of tissue-specific progenitor cells, or some combination of all of these.[3], [39], [40]

Although the precise cellular mechanism for the proliferative response remains uncertain, these observations can be collectively interpreted several ways. (1) Epithelial to mesenchymal transitioning (EMT) occurs allowing the cells to migrate from the urothelium into the LP and incorporate into the MP. EMT is an essential component of bladder development such that embryonic bladder mensenchyme does not differentiate into smooth muscle without adjacent urothelium, and embryonic urothelium causes ingrowth of fibroblasts into an acellular matrix.[25], [41], [42], [43] In addition, it has been reported that this EMT process occurs in the liver under several different conditions.[44] Also, in a recent review *Choi and Diehl* discussed the EMT/MET process in regards to liver regeneration and how the balance between the two processes can modulate the outcome of repair/regeneration. When EMT activity surpasses MET activity, repair is primarily fibrogenic, causing liver fibrosis. On the other hand, when the activity of the MET process out weights the activity of EMT the outcome is more of a normal liver regeneration.[45] Thus, it is reasonable to speculate a similar process may occur in bladder regeneration. (2) Similarly, replicating cells in the urothelium at early time points may induce adjacent cells to proliferate via paracrine signaling. Several studies have focused on defining signaling pathways after bladder injury that enhance urothelial and smooth muscle growth, such as keratinocyte growth factor (KGF), epidermal growth factor (EGF), and transforming growth factor (TGF). Bladder regeneration may also involve signaling pathways similar to those observed in these studies.[42], [46], [47], [48], [49] (3) Individual progenitor cells may be preexisting within all layers of the bladder wall but enter the cell cycle at different time points after STC injury. Consistent with this hypothesis, enquires into the existence of a bladder progenitor cell using BrdU-labeled rabbit models suggest that subepithelial, mesenchymal cells can rapidly proliferate, differentiate and integrate into smooth muscle bundles in response to mechanical stress (outlet obstruction) [31] and cryogenic injury.[27] However, investigating the presence of proliferating cells co-labeled with various stem cell markers (Lgr5, CD-34, SSEA-1, c-kit) has proved challenging.[50] (4) Finally, the proliferating mesenchymal cells may be comprised of bone-marrow derived stem cells arriving via blood stream after the injury, a concept that is well described and supports tissue repair.[51], [52] One or, more likely, a combination of these mechanisms may explain the proliferative cellular landscape we have described. We further speculate that the regenerative process initiated by STC may share common features with bladder development and wound healing. Future experiments will focus on elucidating these commonalities and defining the mechanisms behind functional bladder regeneration *in vivo*.

We also conducted initial investigations into the potential role of hedgehog signaling in STC-induced bladder regeneration. The hedgehog pathway is a well-described and evolutionally conserved component of bladder development that is up regulated in response to bladder injury and therefore might be involved in STC-induced bladder regeneration as well.[34], [53] Immunohistochemical staining provides evidence for increased expression of Shh, Gli-1 and BMP-4 at various time points post-STC (Figs. 7 & 8).

In this regard, *Shin et al.* found that Shh expression and cell proliferation increased dramatically throughout the urothelium after bacterial injury to the mouse bladder. This study confirmed Shh activity and up-regulated Gli-1 staining within 24 hours of the injury, similar to our results.[34] Gli-1 is a downstream transcription factor of Shh (eg, indicator of Shh activity) that activates the Wnt gene family and BMPs to promote cellular differentiation.[25] Furthermore, *Cao et al.* highlighted the importance of Shh signaling to mesenchymal-epithelial interactions during proper bladder development in mice with higher concentrations of Shh increasing both the number of cells and bladder size at 12-days gestation. However the presence of Shh without adjacent urothelium did not give rise to appropriate bladder development.[54] The abundance of Shh observed after STC, along with the importance of mesenchymal-epithelial interactions as described by *Baskin* and colleagues suggests that the Shh pathway may be responsible, at least in part, for aspects of functional bladder regeneration.[41], [42], [54]

We also evaluated the expression of BMP-4 after STC. As part of the TGF-β superfamily, BMP-4 is a downstream signaling protein of the Shh pathway mediating SMAD transcriptional factors and promoting entry into the cell cycle.[53] It is a key developmental signal and regulator of urothelial self-renewal following injury.[33] In our current study BMP-4 was most prominent at 3-days post-STC. Taken together, our data provides initial evidence that the hedgehog pathway may play an important role in functional bladder regeneration.

In conclusion, this initial study describes early quantitative and time-dependent changes in the location of DNA synthesizing/proliferating cells that occur with the corresponding increased expression of several components of the hedgehog signaling pathway during complete structural and functional bladder regeneration in the rat. Presumably, this proliferative response explains the rapid increase in bladder size and structural/functional recovery after STC, as demonstrated in our previous study.[14] Understanding the cellular and molecular mechanisms responsible for the functional bladder regeneration observed in this rodent model may have important implications for more widespread clinical applications of regenerative medicine and tissue engineering technologies.
